# Associations of Polyunsaturated Fatty Acid Intake with Bone Mineral Density in Postmenopausal Women

**DOI:** 10.1155/2015/737521

**Published:** 2015-02-17

**Authors:** Margaret Harris, Vanessa Farrell, Linda Houtkooper, Scott Going, Timothy Lohman

**Affiliations:** ^1^Department of Health Sciences, University of Colorado Colorado Springs, 1420 Austin Bluffs Parkway, Colorado Springs, CO 80918, USA; ^2^Department of Nutritional Sciences, University of Arizona, 1177 E. 4th Street, Tucson, AZ 85721, USA; ^3^Nutritional Sciences Department, College of Agriculture and Life Sciences, University of Arizona, Tucson, AZ 85721-0036, USA; ^4^Department of Physiology, 3017 N. Gaia Place, Tucson, AZ 85745, USA

## Abstract

A secondary analysis of cross-sectional data was analyzed from 6 cohorts (Fall 1995–Fall 1997) of postmenopausal women (*n* = 266; 56.6 ± 4.7 years) participating in the Bone Estrogen Strength Training (BEST) study (a 12-month, block-randomized, clinical trial). Bone mineral density (BMD) was measured at femur neck and trochanter, lumbar spine (L2–L4), and total body BMD using dual-energy X-ray absorptiometry (DXA). Mean dietary polyunsaturated fatty acids (PUFAs) intakes were assessed using 8 days of diet records. Multiple linear regression was used to examine associations between dietary PUFAs and BMD. Covariates included in the models were total energy intake, body weight at year 1, years after menopause, exercise, use of hormone therapy (HT), total calcium, and total iron intakes. In the total sample, lumbar spine and total body BMD had significant negative associations with dietary PUFA intake at *P* < 0.05. In the non-HT group, no significant associations between dietary PUFA intake and BMD were seen. In the HT group, significant inverse associations with dietary PUFA intake were seen in the spine, total body, and Ward's triangle BMD, suggesting that HT may influence PUFA associations with BMD. This study is registered with clinicaltrials.gov, identifier: NCT00000399.

## 1. Introduction

Experts predict that osteoporosis-related fractures can increase health care costs approximately $25.3 billion by 2025 [1]. Diet is a key lifestyle factor that can modify risk and facilitate the prevention of osteoporosis [2]. The amount and type of fat consumed have been linked to bone loss [3, 4]. Of the different types of fats, polyunsaturated fatty acids (PUFAs) are receiving recognition for having varying roles in the prevention and treatment of osteoporosis [2–7].

Among the PUFAs, omega-3 fatty acid (n-3 FA) and omega-6 FA (n-6 FA) are the primary precursors of the eicosanoids (the signaling molecules), which modulate intracellular signal transduction and cell-to-cell interactions [2]. It has been suggested that the eicosanoids, derived from n-6 FA, promote cell proliferation and inflammation which can affect bone resorption negatively; eicosanoids from n-3 FA promote anti-inflammatory action and thereby inhibit bone resorption [8, 9].

During perimenopause, bone density starts to drastically decrease with the decreasing amounts of estrogen. However, HT can slow the decrease in bone density and may even be used as a treatment for postmenopausal osteoporosis. Two studies that examined the relationship between PUFA, bone and HT, found that the relationship between PUFA and bone may be modified dependent on hormone therapy use, though the findings have not been consistent [10, 11].

To date, in vitro and animal studies support n-3 FA to have beneficial effects on bone. Human studies are limited in number and have resulted in conflicting evidence [7]. Limitations included study design and nonhomogenous populations. The purpose of this analysis was to determine whether the dietary PUFA intakes and the ratios of specific PUFAs have associations with femur neck, trochanter, lumbar spine, Ward's triangle, and total body BMD in a sample of 266 postmenopausal women. Second, the relationships between BMD and dietary PUFA intakes and their ratios categorized by HT use were investigated.

## 2. Methods

### 2.1. Design

This is a secondary analysis of cross-sectional data that was collected during the first year of the Bone Estrogen Strength Training (BEST) study, a blocked-randomized, clinical trial. The BEST study investigated the effect of exercise on BMD in postmenopausal women [12]. Participants were categorized by use of HT and then randomized to exercise or control conditions. Participants were instructed to consume 800 mg of calcium supplements daily (provided by the study) during the trial to minimize variability in calcium intake. The University of Arizona Internal Review Board approved the study and the subjects provided written and informed consent.

### 2.2. Subject Entry Criteria

The BEST study enrolled 6 cohorts of women (Fall 1995–Fall 1997) who met the following inclusion criteria: 40–65 years of age; surgical or natural menopause (3.0–10.9 years); body mass index (BMI) >19.0 kg/m^2^ and <32.9 kg/m^2^; nonsmoker; no history of osteoporotic fracture and an initial BMD greater than *Z*-score of –3.0 at all bone sites of interest; taking HT (1.0–5.9 years) or not taking HT (>1 year); weight gain or loss ≤13.6 kg in the previous year; cancer and cancer treatment-free ≥5 years (excluding skin cancer); not taking BMD-altering medications, beta-blockers, or steroids; dietary calcium intake >300 mg/day; performing <120 minutes of low intensity, low impact exercise per week and no weightlifting or similar physical activity. Participants agreed to accept randomization to exercise or no-exercise groups, continue their baseline level of physical activity (if not randomized to exercise), continue their usual dietary practices, maintain their HT status, and take 800 mg of calcium supplements each day of the trial [12, 13].

Three hundred twenty-one women were enrolled in the primary study. The current analyses with one-year data excluded 55 participants: women who had <5 days of diet records (DR) or did not have valid year-one dual-energy X-ray absorptiometry (DXA) measurements. The final sample for analysis included 266 women.

### 2.3. Anthropometry

Trained anthropometrists took three measurements of each variable at each assessment (baseline, 6 months, and 12 months), which were averaged to obtain the criterion measures. Twelve-month means were used in these analyses. Subjects wore lightweight clothing without shoes for measurements of height and weight. Standing height was measured to the nearest 0.1 cm during a maximal inhalation using a Schorr measuring board (Schorr Products, Olney, MD). Weight was measured on a calibrated digital scale (SECA, model 770, Hamburg, Germany) accurate to 0.1 kg. Body mass index (BMI) in kilograms per meter squared was calculated from weight (kg) and height (m).

### 2.4. Dual-Energy X-Ray Absorptiometry

The DXA was used to measure femur neck and trochanter, lumbar spine (L2–L4), and total body BMD (Lunar, Model DPX-L; software version 1.3y, extended research analysis, pencil beam densitometer, Lunar Radiation Corp, Madison, Wisc., USA). Standardized data acquisition and analysis techniques were used [12]. Each subject was scanned twice at each measurement period (baseline, 6 months, and 12 months) and the mean of the two measurements taken at 12 months was used in this analysis. Soft tissue composition was also derived from DXA whole body scans.

### 2.5. Diet Assessment

Dietary intake was assessed from eight randomly assigned days of diet records (DR) collected at baseline (3 days), 6 months (2 days), and 12 months (3 days). Diet data was averaged over a year to provide a more stable and representative average of dietary intake; hence a 12-month BMD estimate rather than baseline was utilized. Participants completed an intensive 1.5 h of DR training prior to each recording period. Training consisted of participatory portion size and dimension estimation, directions on recording food descriptions, and evaluation of portion size estimation accuracy [14]. Participants did not receive dietary advice and were instructed to refrain from changing their diets during the study. Each 2- to 3-week recording period included one weekend day and 1-2 nonconsecutive, random weekdays. Seasonal eating, consecutive day food leftovers, and weekend eating were taken into account by assessing intake at three time points, at which one day per week was recorded over a 2- to 3-week period throughout the year.

Completeness and accuracy of the DR were fostered by personal interviews given by trained technicians. Recipes, labels, and restaurant information were collected to enhance food item entry. The DR were analyzed for estimated mean nutrient intakes using the Minnesota Nutrient Data System (NDS) 93 (versions 2.8–2.92, 1995–1999, Nutrition Coordinating Center, Minneapolis, MN). Foods not in the database were substituted with a similar food item that was within 90% agreement for energy, carbohydrate, protein, fat, and sodium of the original food. A master control process for each cohort, by test period, tracked each DR through the data entry process. This process included initial entry of the data, checking the NDS analysis with the original DR, correcting any errors to the data, checking the corrections, final corrections, and filing of completed records [15].

Every two months, participants received blister packs of calcium citrate tablets (Citracal, Mission Pharmacal, San Antonio, TX). Instructions on calcium supplement intake were given at each of the DR training sessions. The subjects were instructed to take 2 tablets (200 mg elemental calcium/tablet), twice a day (800 mg/day), without food, with a minimum of 4 hours between doses. Calcium supplement compliance was monitored through tablet counts. Participants were considered compliant if they consumed at least 80% of their calcium tablets. Efficacy of adequate calcium intake from diet and supplementation to improve bone health has been previously reported for this population [16–18]. Calcium supplementation was evaluated in this investigation as a potential confounder of the primary analysis regarding dietary PUFA intake associations with BMD.

Iron and calcium intakes have been shown to have a relationship with BMD in this sample [17, 18]. Because of these previous associations, calcium from diet only, total calcium, iron from diet only, and total iron were included in the analysis. Total calcium intake was calculated as the sum of the mean calcium intake obtained from the diet only and the mean intake from the calcium supplements calculated through tablet count compliance. Total iron intake was calculated as the sum of the mean iron intake obtained from the diet only and the mean of any supplemental iron recorded in the DR. Total calcium and total iron are the only variables that include supplemental intake in this analysis.

### 2.6. Statistical Methods

All data analyses were performed using the Statistical Package for the Social Sciences (SPSS, version 21). Mean nutrient intake values were calculated from estimates of dietary intake only except for total calcium and total iron (which included dietary and supplemental intakes). The independent variables included dietary: total fat, total PUFA, all n-6 FA, linoleic acid (LA), arachidonic acid, all n-3 FA, EPA + DHA, *α*-linolenic acid (ALA), the ratios n-6 FA : n-3 FA, and LA : ALA. The women in this sample were not supplemented with omega-3. The dependent variables included BMD from femur neck, Ward's triangle, trochanter, lumbar spine (L2–L4), and total body BMD.

Descriptive characteristics for body composition and mean nutrient intakes from DR were calculated. Student's *t*-tests at one year were used to detect statistically significant differences in mean nutrient intakes between the subjects who used HT and those who did not use HT. Nutrient intake distributions were examined and transformed (log and square root), when appropriate, to meet the assumptions of normality of the statistical tests. Pearson's correlations were computed between all nutrients and covariates at one year. Separate multiple linear regressions were used to examine the dietary PUFA associations with BMD. The multiple linear regression model used with the total sample included three* a priori* coded contrasts that were included as covariates: contrast 1 = exercise versus no exercise within HT; contrast 2 = exercise versus no exercise within no-HT groups; HT contrast 3 = HT versus no HT. Other covariates used in multiple regression included year 1 weight, years after menopause, total energy intake, calcium, and iron. The sample was also stratified by HT use since this block design was selected from two different samples of women based on previous HT use. Covariates for this analysis included energy intake, exercise, year 1 weight, years after menopause, total calcium, and total iron. The relationship between PUFA and bone was further characterized by creating tertiles for omega-6 and omega-3 nutrients, plotted against total body BMD, while adjusting for covariates using the general linear model. Significance was evaluated at the *P* < 0.05 level. With *n* = 266, power is ≥0.99 for all adjusted *R* ^2^.

## 3. Results

Table 1 summarizes twelve-month subject characteristics for 266 postmenopausal women as a whole group then by HT status. Women not using HT were older, had been menopausal longer, and had significantly lower BMD at all bone sites compared to women on HT. Based on BMI, participants on average were slightly overweight (25.7 ± 3.9). Although mean values were reported for one year, women had not lost or gained weight over the course of the year since the start of the study.

The one-year significant nutrient intake associations with BMD using multiple linear regression analyses are summarized in Table 2. Iron intake and HT use were significant confounding factors and strong predictors of BMD at each site in multiple regression models. Significant negative associations between PUFA intake of total fat, total PUFA, omega-6, LA, omega-3, LA, and ALA and BMD were found for the lumbar spine and total body. These PUFAs were not associated with BMD at the femoral neck, Ward's triangle, or trochanter. Other PUFAs (arachidonic acid, EPA + DHA, n-6 : n-3, and LA : ALA) showed no association with bone at any site (data not shown), except for arachidonic acid, which showed a small positive association with trochanter only (adjusted *β* = 0.134, *P* = 0.03).

This study further determined that the relationship between mean PUFA intakes and BMD would vary with HT use. In the no-HT group, a significant, positive association between arachidonic acid and trochanter BMD was found (*β* = 0.181, *P* = 0.04). No other significant associations were found between PUFAs and bone at any site in the non-HT group.  Table 3 summarizes the significant findings between the PUFAs and bone that were significant (by HT). In the HT group, significant inverse associations were seen with total fat, total PUFA, n-6 FA, LA, n-3 FA, and ALA at the lumbar spine BMD, Ward's triangle, and total body (n-3 FA and ALA only). Femur neck was not associated with any PUFA.

**Figure 1:**
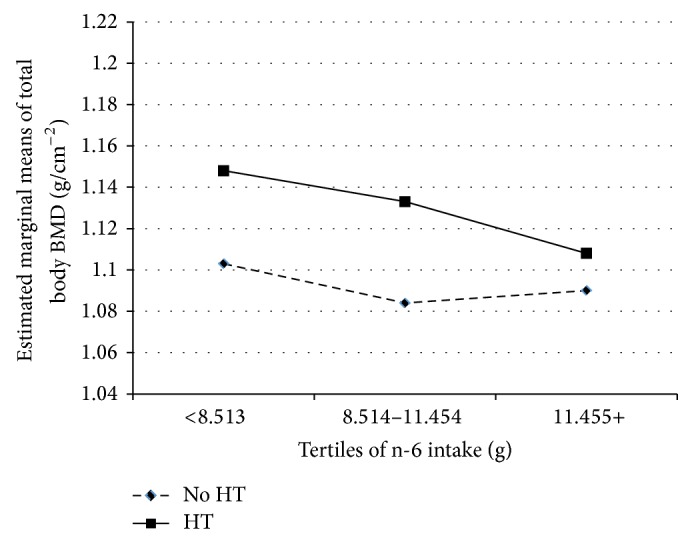
Associations of n-6 with total body BMD (g/cm^2^). Covariates included energy intake, calcium intake, iron intake, years after menopause, and exercise.

**Figure 2:**
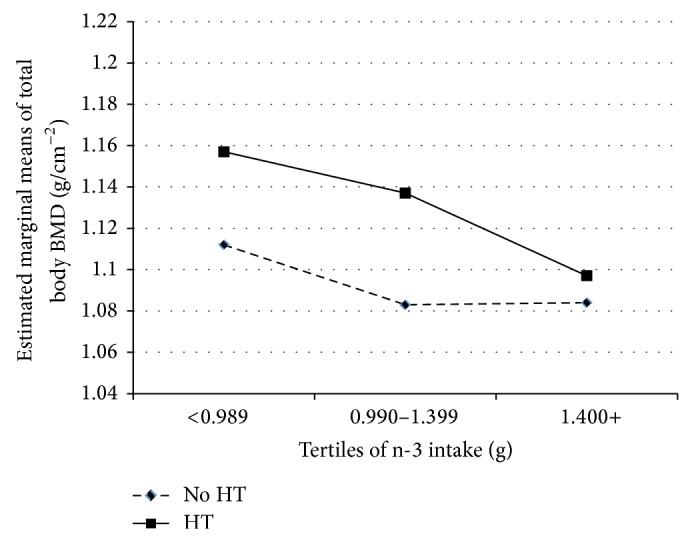
Associations of n-3 with total body BMD (g/cm^2^). Covariates included energy intake, calcium intake, iron intake, years after menopause, and exercise.

To illustrate the relationship between PUFA and bone, tertiles of n-6 and n-3 FA were plotted by HT against total body BMD (adjusting for exercise, weight, years after menopause, total energy, calcium, and iron intake) are presented (Figures 1 and 2). Women who used HT had greater bone density than women who did not use HT, even after adjusting for covariates. However, increasing intake of both n-6 FA and n-3 FA showed a progressive and significant (*P* ≤ 0.05) downward trend for total body BMD.

## 4. Discussion

This cross-sectional analysis showed that PUFA intakes and their ratios assessed by repeat DR over a 12-month period were significantly associated with BMD at the lumbar spine, total body, and Ward's triangle in postmenopausal women though the associations differed by PUFA and HT. No association with dietary PUFA and femur neck BMD was observed in any of these analyses. The ratios of the PUFAs also showed no association with BMD. It was determined that the relationships between some dietary PUFA intakes were seen in the HT group but not in the non-HT group (with the exception of arachidonic acid which was positively associated with bone at the trochanter). This study sample was unique in the analysis because of the stratifications of the results by HT use. In 2002, the Women's Health Initiative trial reported increased risk of coronary events and breast cancer in women on HT [19]. However, the BEST study was conducted before these results were released, providing an opportunity to study bone-nutrient interactions with hormone therapy.

Several studies have examined habitual dietary intake of total PUFA and the relationship with BMD in women, though results have been conflicting [10, 11, 20, 21]. In a study in 891 women (50–59 y), not grouped by HT use, Macdonald et al. [20] reported that greater total PUFA intake, assessed by food frequency questionnaire (FFQ), was associated with greater BMD loss at the femur neck particularly among women with lower calcium intakes. No significant associations were observed in relation to spine BMD. In comparison, the current analysis did not find any significant associations with PUFA intake and femur neck BMD. However, the current analysis did demonstrate significant inverse associations between dietary PUFA intake with spine and total body BMD. In addition, while Macdonald et al. [20] only examined total PUFA, this analysis also showed similar negative trends across other specific PUFAs (n-6 FA and n-3 FA, ALA, LA, total PUFA and total fat). The Rancho Bernardo study (*n* = 890 postmenopausal women) found that a higher ratio of n-6 FA : n-3 FA (7.9 ± 2.2 g or ~10 : 1), assessed by FFQ, was negatively associated with BMD at the hip and at the spine in women using HT and not using HT (though the results were not significant in women taking HT on spine) [10]. In contrast, the current analysis did not show the ratio of n-6 FA : n-3 FA (8.16 ± 2.2 g or ~10 : 1) to be significantly associated with BMD at any bone site nor by HT status though we did consistently show negative associations with other PUFAs and bone sites. The OSTPRE Fracture Prevention Study (*n* = 544) [11] also showed HT to modify the effect of PUFA with bone but in opposition to this study's findings: women not taking HT had positive relationships with PUFAs and spine BMD but not femoral neck but women taking HT showed no relationships with PUFAs. These women were younger than those in this study, suggesting age can also modify the relationship with bone even within an older age group.

Although the negative associations between n-6 FA, LA, and bone were expected due to their inflammatory nature, this study did not expect negative associations with n-3 FA, ALA, and bone. Animal studies have suggested a beneficial effect of n-3 FAs on bone health [6], but human studies have not been as consistent. Since dietary intake sources of n-3 FAs tend to be low in this study and in general populations, it is possible that a much higher intake of n-3 FAs is necessary to see the positive effects on bone.

One way to observe this is through supplementation. There have been only a few dietary intervention studies in postmenopausal women investigating the role of supplemental intake of PUFA in osteoporosis and these studies have also yielded equivocal results, although the forms of supplementation varied ranging from PUFA [22], DHA, and EPA [23] and evening primrose oil [24–26]. The results from these studies are difficult to compare due to variations in sample size, age of participants, dose of PUFA, type of PUFA, length of interventions, and methods of assessing the independent nutrient intake variables, as well as the diet and baseline bone health of the sample population. These factors likely influence the variability of the results of these studies. This current analysis was not a dietary intervention and only presented results cross sectionally and therefore could not establish causality. It does, however, add to the limited body of research investigating the roles of dietary PUFAs and bone health, including modification by HT.

The optimal ratio of n-6 FA : n-3 FA for bone health is unknown. Simopoulos has suggested that within the last 100 years Western's society has changed the dietary n-6 FA : n-3 FA intake ratio from 1 : 1 to 15 : 1 postulating that the current Western diet is “proinflammatory” and may lack the optimal quantity of n-3 FA to promote bone health [27]. These analyses showed a 10 : 1 ratio of n-6 FA : n-3 FA, thus indicating this sample of postmenopausal women's overall dietary intake of PUFA may not be optimal to promote bone health. A low intake ratio of n-6 to n-3 fatty acids appears to decrease the risk of osteoporosis and slow the rapid rate of postmenopausal bone loss [4, 8]. It is also possible that consumption of PUFAs from various sources (marine versus plant) may absorb differently and contribute to conflicting results. The Dietary Reference Intake of n-3 FA for women 14 years and older is 1.1 grams/day, while the Acceptable Macronutrient Distribution Range (AMDR) is 0.6% to 1.2% of total energy [28]. In this study of postmenopausal women, n-3 FAs were slightly higher than the recommendation for women (1.30 ± 0.5 grams) but the women were also predominantly “meat eaters” suggesting a diet more prone towards inflammation.

This study is intriguing and supports the notion that PUFA impact on bone is modified by HT. Although the spine, Ward's triangle, and total body had negative associations with PUFA in both HT and non-HT using women, the relationships were statistically significant only in the HT women. Although the error was similar, the slopes of the regression lines were steeper in the HT group suggesting a stronger relationship. One possible explanation is the modifying impact of exercise. In this study the average bone mineral density was higher in women who exercised versus those who did not exercise, particularly in those who exercised and used HT, though, in statistical analyses, exercise was not a significant confounder in the PUFA, bone, and HT relationship. Another explanation is the complication of overall diet. Some studies have suggested that fatty acids can interact with sex hormones, affecting nutrient absorption and fatty acid status, as can the type of HT (estrogen versus estrogen and progesterone) [29–31]. How various types of HT impacted bone was not evaluated. Vitamin D status or intake which is known to be a significant nutrient in its ability to influence the absorption of other bone building nutrients [32] was also not evaluated.

This study was limited by the lack of biological markers of bone turnover for comparison with other studies to understand this relationship fully. The nutrient database used in the analysis is the most comprehensive database for dietary fatty acids but did contain incomplete or missing nutrient values for some foods. Dietary assessment methods have their limitations even with frequent updates of food nutrient compositions; however, both report and systemic biases introduced are expected to be equivalent across groups. Further, biologically it is unclear why these analyses found n-6 FA and in particular n-3 FA would have an inverse relationship with spine and total body BMD and why AA acid would have a positive association with femur trochanter BMD. One possible explanation may be that sixteen to twenty percent of the values of these nutrients, LA, ALA, n-6 FA, or n-3 FA, are estimated values in NDS nutrient database. In addition, the PUFA intake was solely from diet during a time when populations were not yet supplementing with PUFA's, particularly omega-3s. It is possible that the beneficial effects of omega-3 that some research has found require much higher (i.e., supplemented) doses than what this population consumed from solely dietary sources. Another explanation may be that the PUFAs only offer mild effects on bone metabolism [33], and it is still not understood whether PUFAs have varying effects on different bone sites. Also, the overall diet tended to be inflammatory in nature with an excess of n-6 compared to n-3. As Kajarabille et al. suggested, particular focus should be placed on determining effects from supplements from marine animals (i.e., fish oils, krill oils, and cod liver oils) compared to plant-based sources (flaxseed oil, flaxseeds, chia seeds, and primrose oil) as these are very popular today for both heart disease prevention and cognitive function [33].

This study's strength was the extensive 8 days of DR collected and analyzed with a nutrient database that included extensive fatty acid analyses of foods. This study examined five bone sites compared to many studies which were more limited in the number of bone sites examined. Although there have been larger studies associating PUFA's with BMD, they have used the FFQ to assess nutrient intake, which is not as sensitive as the DR [34]. Research is emerging showing that different nutrients have associations at different BMD sites [17, 18, 35]. Future research needs more controlled, randomized, and blinded dietary and supplemental trials to augment these findings. The focus should be on which PUFAs, the amount of PUFAs and to establish the effect of PUFAs on specific bone sites. Out study suggests that HT and dietary PUFA intake are modulators of BMD. With more conclusive dietary studies, especially those that investigate cause and effect, dietary recommendations can aid in the prevention and treatment of osteoporosis.

## 5. Conclusion

This cross-sectional study among 266 postmenopausal women found dietary intakes of PUFA, n-6 FA, LA, n-3 FA, and ALA had significant inverse associations with lumbar spine and total body BMD. Arachidonic acid had the only positive association, which was at the trochanter BMD. When stratified by HT use, PUFA associations with BMD remained significant in the HT group but were lost in the no-HT group. In the HT group, n-3 FA, ALA had an inverse relationship with spine and total body BMD and LA : ALA had a positive relationship with total body BMD. These results suggest that PUFA's effects on bone are modulated by hormone therapy.

## Figures and Tables

**Table 1:** One-year characteristics of 266 postmenopausal women using or not using hormone therapy (HT).

Characteristics	All women (*n* = 266)	HT status	
		HT (*n* = 136)	No HT (*n* = 130)
Age (years)	56.7 ± 4.7	55.9 ± 4.4	57.5 ± 5.0^a^
Height (cm)	163.2 ± 6.5	163.3 ± 7.0	163.0 ± 6.1
Weight (kg)	68.4 ± 11.8	68.2 ± 11.8	68.6 ± 11.7
BMI (kg/m^2^)	25.6 ± 3.9	25.5 ± 4.2	25.7 ± 3.7
Years HT	1.4 ± 1.6	2.8 ± 1.1	0.00^c^
Years postmenopausal	5.7 ± 3.0	4.9 ± 2.5	6.5 ± 3.1^c^
Nutrient variables			
Energy (kcals)	1704 ± 361	1734 ± 372	1672 ± 349
Total fat (g)	58 ± 21	60 ± 21	56 ± 20
Calcium from diet (mg)	769 ± 258	785 ± 258	753 ± 258
Total calcium (mg)	1524 ± 296	1552 ± 288	1497 ± 302
Iron from diet (mg)	15 ± 5	14 ± 4	15 ± 5
Total iron (mg)	17 ± 9	18 ± 11	16 ± 7
PUFA (g)			
Total PUFA	11.7 ± 4.4	12.11 ± 4.58	11.26 ± 4.25
Total n-6 fatty acid (n-6)	10.4 ± 4.0	10.75 ± 4.12	9.96 ± 3.82
Linoleic acid, n-6 (LA)	10.2 ± 4.0	10.60 ± 4.13	9.78 ± 3.84
Arachidonic acid, n-6 (AA)	0.10 ± 0.1	0.10 ± 0.05	0.11 ± 0.06
Total n-3 fatty acids (n-3)	1.30 ± 0.5	1.29 ± 0.55	1.23 ± 0.50
*α*-Linolenic acid, n-3, (ALA)	1.10 ± 0.5	1.17 ± 0.53	1.11 ± 0.48
EPA	0.05 ± 0.07	0.04 ± 0.06	0.05 ± 0.08
DHA	0.11 ± 0.15	0.10 ± 0.12	0.12 ± 0.17
EPA + DHA	0.16 ± 0.21	0.14 ± 0.17	0.18 ± 0.25
n-6 : n-3	8.45 ± 1.87	8.62 ± 1.98	8.27 ± 1.74
LA : ALA	9.30 ± 2.20	9.47 ± 2.28	9.15 ± 2.04
Bone mineral density (g/cm^2^)			
Femur neck	0.878 ± 0.122	0.896 ± 0.123	0.858 ± 0.118^b^
Ward's triangle	0.760 ± 0.142	0.778 ± 0.137	0.742 ± 0.146^a^
Femur trochanter	0.751 ± 0.113	0.767 ± 0.113	0.733 ± 0.112^a^
Lumbar spine L2–L4	1.133 ± 0.160	1.164 ± 0.143	1.101 ± 0.171^c^
Total body	1.112 ± 0.084	1.129 ± 0.078	1.095 ± 0.086^c^

PUFA: polyunsaturated fatty acid; ALA: *α*-linolenic acid (18 : 3 n-3); total n-3: total omega-3; LA: linoleic acid (18 : 2 n-6); AA: arachidonic acid (20 : 4 n-6); total n-6: total omega-6; EPA: eicosapentaenoic acid (20 : 5 n-3); and DHA: docosahexaenoic acid (22 : 6 n-3).

^a^ *P* ≤ 0.05, ^b^ *P* ≤ 0.01, and ^c^ *P* ≤ 0.001, between HT and no HT, independent sample *t*-test.

**Table 2:** Significant one-year nutrient associations with bone mineral density using multiple regression analysis in 266 postmenopausal women.

	Total body BMD g/cm^2^		Spine (L2–L4) BMD g/cm^2^	
	Std *β*	*P* value	Std *β*	*P* value
Total fat	−0.221	0.055	−0.256	0.032
Total PUFA	−0.157	0.050	−0.192	0.021
Total n-6 FA	−0.158	0.070	−0.187	0.039
Linoleic acid	−0.157	0.072	−0.189	0.037
Total n-3 FA	−0.200	0.008	−0.168	0.032
*α*-Linolenic acid	−0.226	0.002	−0.185	0.017

Covariates used in multiple regression: weight at year 1, years after menopause, HT status, exercise status within HT, total energy intake, calcium, and iron.

Note: arachidonic acid was significant at the trochanter only, standardized *β* = 0.134, *P* value = 0.03.

**Table 3:** One-year nutrient associations with bone mineral density by hormone therapy using multiple regression analysis in postmenopausal women taking hormone therapy.

	HT+ (*n* = 136)					
	Total body		Spine (L2–L4)		Ward's triangle	
	Std *β*	*P* val	Std *β*	*P* val	Std *β*	*P* val
Total fat	−0.464	0.006	−0.460	0.007	−0.341	0.040
Total PUFA	−0.241	0.040	−0.255	0.039	−0.274	0.011
Total n-6 FA	−0.231	0.060	−0.287	0.018	−0.285	0.017
Linoleic acid	−0.232	0.060	−0.260	0.017	−0.282	0.019
Total n-3 FA	−0.302	0.005	−0.260	0.017	−0.258	0.016
*α*-Linolenic acid	−0.241	0.040	−0.460	0.007	−0.341	0.040

Note: women not taking HT had no significant associations with bone sites.
